# A 2-Biomarker Model Augments Clinical Prediction of Mortality in Melioidosis

**DOI:** 10.1093/cid/ciaa126

**Published:** 2020-02-08

**Authors:** Shelton W Wright, Taniya Kaewarpai, Lara Lovelace-Macon, Deirdre Ducken, Viriya Hantrakun, Kristina E Rudd, Prapit Teparrukkul, Rungnapa Phunpang, Peeraya Ekchariyawat, Adul Dulsuk, Boonhthanom Moonmueangsan, Chumpol Morakot, Ekkachai Thiansukhon, Direk Limmathurotsakul, Narisara Chantratita, T Eoin West

**Affiliations:** 1 Division of Pediatric Critical Care Medicine, Department of Pediatrics, University of Washington, Seattle, Washington, USA; 2 Department of Microbiology and Immunology, Faculty of Tropical Medicine, Mahidol University, Bangkok, Thailand; 3 Division of Pulmonary, Critical Care, and Sleep Medicine, Department of Medicine, University of Washington, Seattle, Washington, USA; 4 Mahidol Oxford Tropical Medicine Research Unit, Faculty of Tropical Medicine, Mahidol University, Bangkok, Thailand; 5 Department of Critical Care Medicine, University of Pittsburgh, Pittsburgh, Pennsylvania, USA; 6 Department of Internal Medicine, Sunpasitthiprasong Hospital, Ubon Ratchathani, Thailand; 7 Department of Microbiology, Faculty of Public Health, Mahidol University, Bangkok, Thailand; 8 Department of Medicine, Mukdahan Hospital, Mukdahan, Thailand; 9 Department of Medicine, Udon Thani Hospital, Udon Thani, Thailand; 10 Department of Tropical Hygiene, Faculty of Tropical Medicine, Mahidol University, Bangkok, Thailand

**Keywords:** biomarkers, melioidosis, sepsis, IL-6, IL-8

## Abstract

**Background:**

Melioidosis, infection caused by *Burkholderia pseudomallei*, is a common cause of sepsis with high associated mortality in Southeast Asia. Identification of patients at high likelihood of clinical deterioration is important for guiding decisions about resource allocation and management. We sought to develop a biomarker-based model for 28-day mortality prediction in melioidosis.

**Methods:**

In a derivation set (N = 113) of prospectively enrolled, hospitalized Thai patients with melioidosis, we measured concentrations of interferon-γ, interleukin-1β, interleukin-6, interleukin-8, interleukin-10, tumor necrosis factor-ɑ, granulocyte-colony stimulating factor, and interleukin-17A. We used least absolute shrinkage and selection operator (LASSO) regression to identify a subset of predictive biomarkers and performed logistic regression and receiver operating characteristic curve analysis to evaluate biomarker-based prediction of 28-day mortality compared with clinical variables. We repeated select analyses in an internal validation set (N = 78) and in a prospectively enrolled external validation set (N = 161) of hospitalized adults with melioidosis.

**Results:**

All 8 cytokines were positively associated with 28-day mortality. Of these, interleukin-6 and interleukin-8 were selected by LASSO regression. A model consisting of interleukin-6, interleukin-8, and clinical variables significantly improved 28-day mortality prediction over a model of only clinical variables [AUC (95% confidence interval [CI]): 0.86 (.79–.92) vs 0.78 (.69–.87); *P* = .01]. In both the internal validation set (0.91 [0.84–0.97]) and the external validation set (0.81 [0.74–0.88]), the combined model including biomarkers significantly improved 28-day mortality prediction over a model limited to clinical variables.

**Conclusions:**

A 2-biomarker model augments clinical prediction of 28-day mortality in melioidosis.

## Teaser

Melioidosis is increasingly recognized as a cause of sepsis in tropical regions with high associated mortality. We developed and subsequently validated an inflammatory biomarker-based model for 28-day mortality prediction in melioidosis.

Melioidosis is an often-severe infectious disease caused by the gram-negative bacterium *Burkholderia pseudomallei*. A Centers for Disease Control and Prevention Tier 1 select agent found in tropical soil and water, *B. pseudomallei* is endemic to parts of Southeast Asia as well as northern Australia, yet is increasingly identified throughout the tropics [1–3]. Although the manifestations of infection are highly variable, melioidosis is frequently characterized by a profound systemic inflammatory response resulting in sepsis and septic shock [4–7]. Despite appropriate antibiotic treatment, mortality ranges from 10% to 40%, with estimated average global mortality in 1 modeling study exceeding 50% [8].

Given the high mortality associated with melioidosis as well as the challenges of tertiary center referral in some endemic areas, the development of early-prediction tools for melioidosis outcome is paramount. Such tools could inform resource allocation and clinical management. Clinical scores, such as the Sequential Organ Failure Assessment (SOFA), reasonably predict sepsis-related mortality in multiple studies and are associated with mortality in Southeast Asian cohorts of patients with infection [9, 10]. However, improving on the predictive capacity of these scores is desirable. Furthermore, obtaining the clinical data that comprise such scores may be challenging in low- and middle-income countries [11]. Biomarkers may therefore be useful in overcoming existing limitations in mortality prediction in melioidosis.

A robust inflammatory response is a hallmark of severe sepsis, including severe melioidosis [12]. Elevations in levels of circulating inflammatory cytokines and other inflammatory pathway proteins have been used to predict mortality in critical illness, including septic shock and severe melioidosis [13–16]. However, broad investigations of the predictive capacity of inflammatory cytokines for mortality in melioidosis are limited. We hypothesized that a plasma biomarker model could predict mortality in patients with melioidosis. Moreover, we hypothesized that a biomarker model could augment or supercede clinical estimates of likely mortality. Deployed in a point-of-care fashion at the bedside, such biomarkers could potentially assist clinicians in decision making, including in resource-limited settings.

## METHODS

### Melioidosis Patient Cohorts

#### Derivation and Internal Validation Sets

Subjects aged 18 years or older admitted to Sunpasittiprasong Hospital, Ubon Rachathani, Thailand, from 2013 through 2017 with suspected infection and 3 systemic manifestations of infection, as proposed by the 2012 Surviving Sepsis Campaign, were prospectively enrolled within the first 24 hours of admission [17]. This cohort, and subsets of it, have previously been described [11, 18, 19]. One hundred and ninety-one individuals with any culture of a clinical specimen positive for *B. pseudomallei* were considered for inclusion in the derivation and internal validation sets of this study. Plasma samples were obtained at the time of enrollment. Clinical data were assessed by the study team or extracted from the medical record and recorded in a case report form. We estimated that analysis of 110 patients would yield 85% power to detect improvement in the area under the receiver operating curve (AUC) relative to an AUC of 0.75, assuming 50% nonsurvivors, log mean cytokine (variance) of 2.5 (0.8) in nonsurvivors, and log mean cytokine (variance) of 1.5 (0.5) in survivors, setting an ɑ equal to .05 [20]. Of the 191 subjects, the first 113 consecutively enrolled subjects were selected for the derivation set and the remaining 78 subjects comprised the internal validation set.

#### External Validation Set

The external validation set was a prospectively enrolled cohort of 161 subjects aged 15 years or older admitted to Udon Thani Hospital, Udon Thani, and Mukdahan Hospital, Mukdahan, Thailand, from 2015 through 2018, who had growth of *B. pseudomallei* in any cultured clinical specimen. Enrollment occurred at the time of pathogen identification. This cohort has been described previously [21]. Plasma samples were obtained at the time of enrollment and clinical data were obtained from patient interview, medical records, and telephone calls.

### Clinical Definitions

In the derivation and internal validation sets, a modified SOFA score was calculated for subjects from presentation until the time of enrollment. The cardiovascular and respiratory components of the SOFA score were modified because vasopressor/inotrope dosages were not captured and because arterial blood gases were rarely performed, as reported previously [18, 22]. For the cardiovascular component of the SOFA score, 2 of 4 points were given for receipt of dobutamine or dopamine and 3 of 4 points were given for receipt of epinephrine or norepinephrine. For the respiratory component of the SOFA score, 2 of 4 points were given for advanced respiratory support (endotracheal intubation and mechanical ventilation) when no arterial blood gas result was available.

For the external validation set, the clinical data obtained at enrollment were insufficient to calculate either a standard SOFA score or a modified SOFA score. Therefore, where indicated, models were adjusted for cardiopulmonary organ failure at the time of enrollment. This was defined as respiratory failure requiring invasive mechanical ventilation or shock requiring an infusion of epinephrine, norepinephrine, dopamine, or dobutamine.

### Biomarker Quantification

In the derivation set, plasma concentrations of interferon-γ (IFN-γ), interleukin (IL)-1β, IL-6, IL-8, IL-10, tumor necrosis factor-ɑ (TNF-ɑ), granulocyte-colony stimulating factor (G-CSF), and IL-17A were measured using an electrochemiluminescent multiplex assay (Meso Scale Discovery). Upper and lower limits of detection were determined by the manufacturer’s software. In the internal validation set, plasma concentrations of IL-6 and IL-8 were measured using the same platform.

In the external validation set, concentrations of IL-6 and IL-8 were determined in plasma using bead-based multiplex assays on Luminex technology (Milliplex MAP kit: HSTCMAG-28K-12 plex, Millipore). The limits of detection for IL-6 were 0.18–750 pg/mL and for IL-8 were 0.31–1250 pg/mL.

Biomarker concentrations are reported as median and interquartile range (IQR). Comparisons between patients who survived or died were performed with the Mann-Whitney *U* test.

### Model Development

Candidate models of mortality prediction were initially developed in the derivation set using combinations of clinical variables such as age, sex, Charlson Comorbidity Index, a modified SOFA score, and selected biomarkers. All biomarkers were log_10_-transformed before analysis. To simplify the prediction model to the fewest number of biomarkers possible, all 8 biomarkers were subjected to logistic regression analysis by least absolute shrinkage and selection operator (LASSO) methodology in which lambda was selected by the Akaike Information Criterion; the selected biomarkers were confirmed using lambda selection based on 5-fold internal cross-validation [23, 24].

The LASSO-selected biomarkers and clinical variables were evaluated as predictors of 28-day mortality by creating logistic regression models. All models were assessed for goodness-of-fit using Hosmer-Lemeshow chi-square analysis, and models were compared using the likelihood ratio (LR) test [25]. Receiver operating characteristic curve analysis was performed to evaluate mortality discrimination. Discrimination ability was further assessed using integrated discrimination improvement analysis (IDI) [26, 27].

Candidate predictive models were subsequently analyzed in a similar manner in the internal validation set and in the external validation set using cardiopulmonary organ failure instead of modified SOFA score in the latter. Analyses were performed using Stata version 14.2 (StataCorp). Two-sided *P* values <.05 were considered significant.

### Human Subjects

Written informed consent was obtained from study participants or their representatives prior to enrollment. The studies were approved by the Sunpasitthiprasong Hospital Ethics Committee (039/2556), the Udon Thani Hospital Ethics Committee (0032.102/318), the Mukdahan Hospital Ethics Committee (MEC 010/59), the Ethics Committee of the Faculty of Tropical Medicine, Mahidol University (MUTM2012-024-01 and MUTM2015-002-01), the University of Washington Institutional Review Board (42988), and the Oxford University Tropical Research Ethics Committee (OXTREC172-12).

## RESULTS

### Inflammatory Biomarkers Are Associated With 28-Day Mortality in a Derivation Set of Patients With Melioidosis

IFN-γ, IL-1β, IL-6, IL-8, IL-10, TNF-ɑ, G-CSF, and IL-17A were measured in plasma samples obtained on study enrollment in the derivation set of 113 patients with melioidosis. The demographics of this set are reported in Table 1. Of these patients, 72 of 113 (64%) were male and 50 of 113 (44%) had diabetes. Seventy-six percent of subjects had a modified SOFA score of 2 or higher and the 28-day mortality was 55%.

**Table 1.** Patient Characteristics by Cohort

	Derivation Set (n = 113)	Internal Validation Set (n = 78)	External Validation Set (n = 161)
Baseline characteristics			
Age in years, median (IQR)	54 (45–64)	55.5 (46–66)	54 (45–64)
Male sex, n (%)	72 (64)	60 (77)	114 (71)
Pre-existing conditions			
Charlson Comorbidity Index, median (IQR)	2 (1–3)	2 (1–3)	2 (1–3)
Diabetes, n (%)	50 (44)	38 (49)	119 (74)
Chronic liver disease, n (%)	3 (3)	4 (5)	3 (2)
Chronic kidney disease, n (%)	15 (13)	11 (14)	31 (19)
Chronic cardiovascular disease, n (%)	6 (5)	5 (6)	7 (4)
Chronic lung disease, n (%)	7 (6)	8 (10)	5 (3)
Cancer, n (%)	1 (1)	2 (2)	5 (3)
Human immunodeficiency virus, n (%)	0	0	4 (2)
Chronic steroids, n (%)	1 (1)	0	7 (4)
Transferred from another facility, n (%)	99 (88)	70 (89)	90 (56)
Day 1 modified SOFA score, median (IQR)	4 (2–7)	5 (2–8)	…
Day 1 modified SOFA score ≥2, n (%)	86 (76)	62 (79)	…
Bacteremia, n (%)	93 (82)	56 (72)	131 (81)
28-Day mortality, n (%)	62 (55)	36 (46)	68 (42)

Abbreviations: IQR, interquartile range; SOFA, Sequential Organ Failure Assessment.

Plasma cytokines were compared between those who died and those who survived to 28 days (Table 2). Concentrations of all 8 cytokines were significantly higher in nonsurvivors than in survivors (*P* < .001 for all).

**Table 2.** Plasma Biomarker Concentrations in Survivors and Nonsurvivors at 28 Days in Derivati on and Internal Validation Sets

	Derivation Set, pg/mL				Internal Validation Set, pg/mL			
Biomarkers	All (n = 113)	Survivors (n = 51)	Nonsurvivors (n = 62)	*P* ^a^	All (n = 78)	Survivors (n = 42)	Nonsurvivors (n = 36)	*P* ^a^
IL-6	252 (33–1980)	41 (16–239)	1938 (132–1980)	<.001	109 (36–1305)	44 (21–110)	948 (118–2080)	<.001
IL-8	111 (11–479)	14 (5–49)	304 (78–1725)	<.001	41 (7–251)	9 (3–41)	181 (55–1729)	<.001
G-CSF	169 (54–4165)	59 (32–209)	1680 (117–11388)	<.001	…	…	…	
IFN-γ	1078 (350–3213)	538 (188–1618)	2027 (875–4780)	<.001	…	…	…	
IL-1β	3.6 (2–12)	2 (2–7)	7 (2–25)	<.001	…	…	…	
TNF-ɑ	27 (12–73)	14 (6–37)	50 (20–103)	<.001	…	…	…	
IL-17A	57 (14–132)	18 (12–74)	75 (37–163)	<.001	…	…	…	
IL-10	23 (34–50)	5 (2–28)	34 (17–65)	<.001	…	…	…	

Data are presented as median (IQR).

Abbreviations: G-CSF, granulocyte-colony stimulating factor; IFN-γ, interferon-γ; IL, interleukin; IQR, interquartile range; TNF-ɑ, tumor necrosis factor-ɑ.

^a^ *P* values for Mann-Whitney *U* test of biomarker level between subjects alive and dead at 28 days.

The relationship between log_10_-transformed plasma cytokine concentration and 28-day mortality was analyzed using logistic regression (Table 3). All 8 cytokines were strongly positively associated with 28-day mortality in unadjusted models (*P*  < .001 for all). These strong relationships persisted when the models were adjusted for age, sex, and Charlson Comorbidity Index. In order to account for clinically determined severity of illness, models were additionally adjusted for modified SOFA score. In these models, all cytokines remained significantly associated with 28-day mortality. IL-6 (odds ratio [OR], 3.62; 95% confidence interval [CI], 1.97–6.66), IL-8 (OR, 3.44; 95% CI, 1.87–6.32), and G-CSF (OR, 2.71; 95% CI, 1.64–4.49) were most significantly associated with mortality (*P* < .001) in the modified SOFA-adjusted model. These data indicated that even accounting for pre-existing risk factors and severity of illness, these cytokines were independent predictors of mortality in melioidosis.

**Table 3.** Association of Biomarkers With 28-Day Mortality in the Derivation Set

	Unadjusted			Adjusted^a^			Modified SOFA-Adjusted^b^		
Biomarker (log_10_)	OR	95% CI	*P*	OR	95% CI	*P*	OR	95% CI	*P*
IL-6	4.43	2.55–7.70	<.001	4.59	2.58–8.17	<.001	3.62	1.97–6.66	<.001
IL-8	4.09	2.40–6.97	<.001	4.37	2.47–7.71	<.001	3.44	1.87–6.32	<.001
G-CSF	2.95	1.86–4.67	<.001	3.13	1.93–5.06	<.001	2.71	1.64–4.49	<.001
IFN-γ	3.03	1.65–5.60	<.001	2.93	1.58–5.41	.001	2.29	1.20–4.31	.01
IL-1β	4.14	1.94–8.81	<.001	4.77	2.12–10.75	<.001	3.65	1.57–8.47	.003
TNF-ɑ	4.54	2.10–9.80	<.001	4.45	2.02–9.79	<.001	2.54	1.10–5.85	.03
IL-17A	4.39	2.02–9.52	<.001	4.79	2.09–10.93	<.001	2.74	1.13–6.68	.03
IL-10	3.33	1.82–6.05	<.001	3.34	1.81–6.18	<.001	2.06	1.05–4.03	.04

Abbreviations: CI, confidence interval; G-CSF, granulocyte-colony stimulating factor; IFN-γ, interferon-γ; IL, interleukin; OR, odds ratio; SOFA, Sequential Organ Failure Assessment; TNF-ɑ, tumor necrosis factor-ɑ.

^a^For the adjusted model, *P* values were determined using logistic regression adjusted for age, sex, and Charlson Comorbidity Index.

^b^ For the modified SOFA-adjusted model, *P* values were determined using logistic regression adjusted for age, sex, Charlson Comorbidity Index, and modified SOFA score.

### Derivation Set Biomarker Model Development

To develop a parsimonious biomarker-based model to predict 28-day mortality in melioidosis, LASSO regression was performed on the 8 cytokines in the derivation set. This procedure selected IL-6 and IL-8. The 2 selected biomarkers had a high degree of collinearity (Pearson’s coefficient = 0.87). As our goal was to develop the most parsimonious model possible, we therefore analyzed IL-6 and IL-8 individually as well as in combination.

To determine the added value of IL-6 and IL-8 in the prediction of outcome, a logistic regression model of clinical assessment of severity of illness that included age, sex, Charlson Comorbidity Index, and modified SOFA score was first evaluated without the cytokines and then after the addition of each cytokine individually (Table 4). The addition of either IL-6 (LR *P* = 7.6 × 10^−6^) or IL-8 (LR *P* = 1.1 × 10^−5^) significantly improved the model.

**Table 4.** Mortality Prediction Using Models of Clinical Variables With or Without IL-6 and IL-8 in the Derivation Set

Model and Variable	OR	95% CI	*P*
Clinical variables			
Age	0.99	.95–1.04	.88
Female sex	2.47	1.01–6.04	.05
Charlson Comorbidity Index	1.12	.75–1.66	.55
Modified SOFA score	1.32	1.16–1.51	<.001
Clinical variables + IL-6^a^			
Age	1.00	.95–1.06	.88
Female sex	2.89	1.07–7.95	.04
Charlson Comorbidity Index	1.06	.69–1.65	.78
Modified SOFA score	1.18	1.02–1.36	.02
IL-6	3.62	1.97–6.66	<.001
Clinical variables + IL-8^b^			
** **Age	0.99	.94–1.05	.87
** **Female sex	2.65	.99–7.12	.05
** **Charlson Comorbidity Index	1.21	.77–1.91	.41
** **Modified SOFA	1.16	.99–1.35	.05
** **IL-8	3.44	1.87–6.32	<.001

Abbreviations: CI, confidence interval; IL, interleukin; OR, odds ratio; SOFA, Sequential Organ Failure Assessment.

^a^Model containing clinical variables and log_10_ IL-6 differed significantly by likelihood ratio test (*P* = 7.6 × 10^−6^) compared with the clinical variable model.

^b^Model containing clinical variables and log_10_ IL-8 differed significantly by likelihood ratio test (*P* = 1.1 × 10^−5^) compared with the clinical variable model.

**Figure 1.**
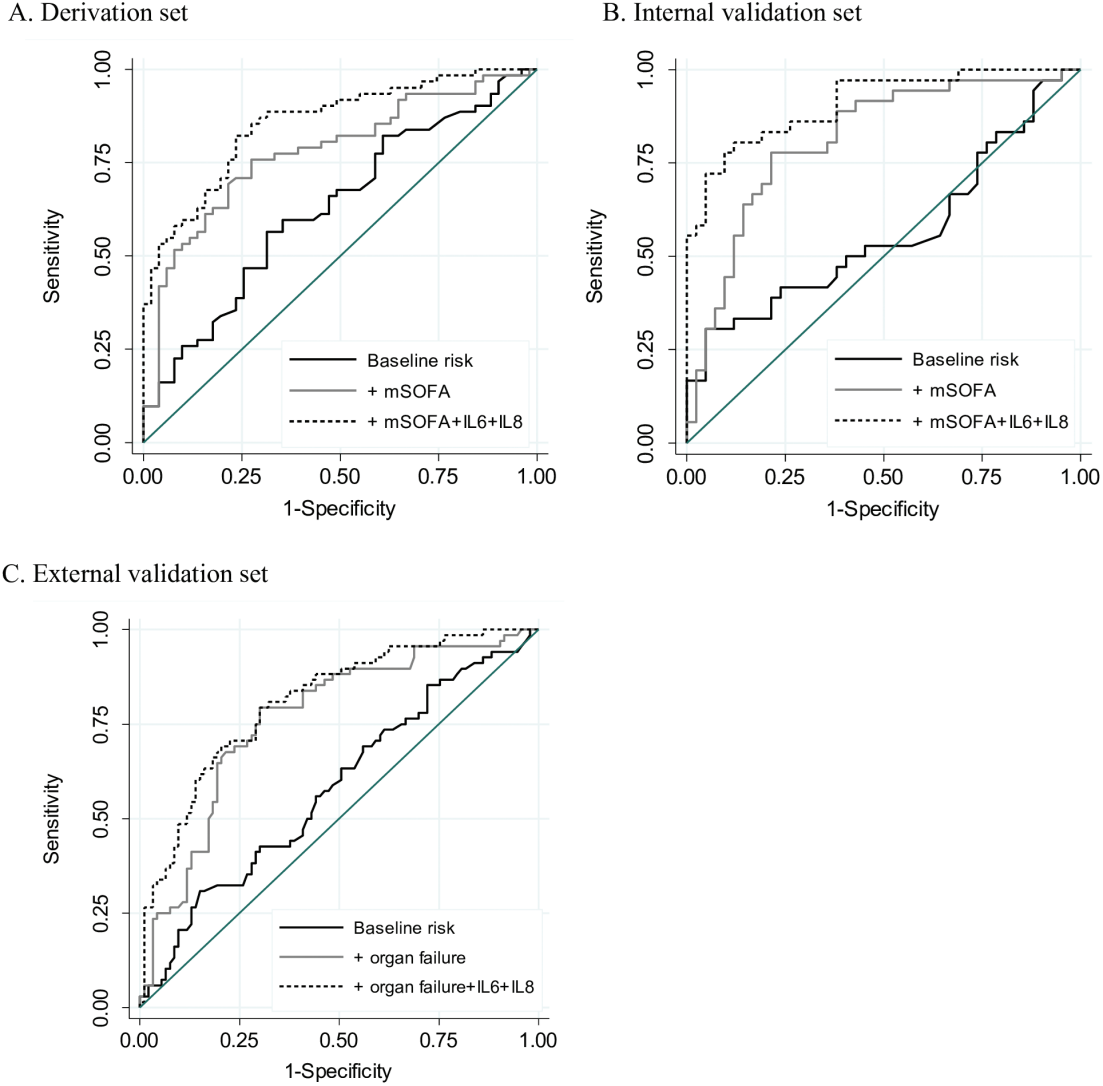
Receiver operating characteristic curves for discrimination of mortality using IL-6 and IL-8. Receiver operating characteristic curves are shown for the baseline risk (age, sex, Charlson Comorbidity Index) model, baseline risk + modified (m) SOFA model, and baseline risk + modified SOFA + IL-6 + IL-8 model for the derivation set (*A*) (AUCs = 0.63, 0.78, and 0.86, respectively) and for the internal validation set (*B*) (AUCs = 0.56, 0.81, and 0.91, respectively). *C*, Receiver operating characteristic curves are shown for the baseline risk (age, sex, Charlson Comorbidity Index) model, baseline risk + cardiopulmonary organ failure (respiratory failure requiring invasive mechanical ventilation or shock requiring an infusion of epinephrine, norepinephrine, dopamine, or dobutamine) model, and baseline risk + cardiopulmonary organ failure + IL-6 + IL-8 model in the external validation set (AUC = 0.58, 0.76, and 0.81, respectively). Abbreviations: AUC, area under the receiver operating curve; IL, interleukin; SOFA, Sequential Organ Failure Assessment.

To evaluate the discrimination of mortality using these biomarkers, receiver operating characteristic curve analysis was performed. The AUC was calculated first for a baseline risk model composed of age, sex, and Charlson Comorbidity Index, and then for models sequentially adding modified SOFA score followed by both IL-6 and IL-8 to the baseline risk model. The model composed of all clinical variables (age, sex, Charlson Comorbidity Index, and modified SOFA score) plus both IL-6 and IL-8 significantly increased mortality discrimination compared with the clinical variable model alone (AUC [95% CI], 0.86 [.79–.92] vs 0.78 [.69–.87]; *P* = .01) (Figure 1A and Supplementary Table 1). Additional discrimination analysis using IDI showed significant improvement in the clinical variable model when IL-6 and IL-8 were added (*P* < .0001) (Supplementary Table 2). The addition of either IL-6 or IL-8 individually to the clinical variable model also significantly improved the discrimination of mortality compared with the clinical variable model alone (Supplementary Figures 1*A* and 2*A*, Supplementary Table 1). Furthermore, a model consisting of both IL-6 and IL-8 without any clinical variables performed similarly to the clinical variable model (AUC, 0.83; 95% CI, .75–.91; *P* = .30) (Supplementary Table 1).

### Internal Biomarker Model Validation

We next analyzed IL-6 and IL-8 as predictors of mortality in the internal validation set of 78 patients with melioidosis. Baseline characteristics of these patients are shown in Table 1. IL-6 and IL-8 were significantly higher in nonsurvivors at 28 days compared with survivors (Table 2) and were significantly associated with 28-day mortality (all *P* < .001) (Supplementary Table 3). The addition of IL-6 and IL-8 to a model of mortality prediction composed of clinical variables was highly significant (LR *P* = 2 × 10^−6^ and 1.5 × 10^−5^, respectively) (Supplementary Table 4). The addition of IL-6 and IL-8 to the clinical variable model significantly improved discrimination of mortality (AUC [95% CI], 0.91 [.84–.97] vs 0.81 [.72–.91], *P* = .03; IDI *P* < .0001) (Figure 1B, Supplementary Tables 1 and 2). The addition of IL-6 and IL-8 individually to the clinical variable model also significantly improved the discrimination of mortality compared with the clinical variable model alone (Supplementary Figures 1*B* and 2*B*, Supplementary Table 1). A model consisting only of IL-6 and IL-8 performed similarly to the clinical variable model (AUC, 0.88; 95% CI, .80–.96; *P* = .20) (Supplementary Table 1).

### External Biomarker Model Validation

As an external validation of our findings, we analyzed the mortality prediction of IL-6 and IL-8 in an independent set of 161 patients with melioidosis enrolled at other sites in northeast Thailand (Table 1). As previously reported, the plasma concentrations of IL-6 and IL-8 were significantly higher in 28-day nonsurvivors than in survivors (median [IQR]: IL-6, 45 pg/mL [20–172] vs 14 pg/mL [7–30], *P* < .001; IL-8, 87 pg/mL [24–199] vs 14 pg/mL [6–36], *P* < .001) [21]. IL-6 and IL-8 each were associated with 28-day mortality in logistic regression models adjusted for age, sex, comorbidities, and cardiopulmonary organ failure (*P* = .003 and *P* = .001, respectively) (Supplementary Table 5). The addition of either IL-6 or IL-8 to a model composed of clinical variables (age, sex, comorbidities, and cardiopulmonary organ failure) significantly improved mortality prediction as determined by the LR (*P* = .002 and 8 × 10^−4^, respectively) (Supplementary Table 6). The addition of IL-6 and IL-8 to the clinical variable model significantly improved discrimination of mortality (AUC [95% CI], 0.81 [.74–.88] vs 0.76 [.68–.84], *P* = .02; IDI *P* = .001) (Figure 1C, Supplementary Tables 1 and 2). The addition of IL-8 but not IL-6 individually to the clinical variable model also significantly improved the discrimination of mortality compared to the clinical variable model alone (Supplementary Figures 1*C* and 2*C*, Supplementary Table 1). A model consisting of only IL-6 and IL-8 performed similarly to the clinical variable model (AUC, 0.78; 95% CI, .71–.85; *P* = .67) (Supplementary Table 1).

## DISCUSSION

In this study of hospitalized patients with melioidosis, we develop and validate parsimonious mortality prediction models comprising plasma IL-6 and IL-8. We report that these biomarkers augment concurrent clinical assessments that include pre-existing comorbidities and markers of organ failure in predicting mortality from melioidosis. Moreover, by themselves, these biomarkers are comparable to clinical models of mortality prediction. Therefore, these biomarkers have the potential to be valuable for clinicians caring for patients with melioidosis in settings with or without the ability to clinically characterize organ failure.

Relatively few prior reports have evaluated the relationship of plasma cytokine concentrations and clinical outcome in melioidosis. In older analyses of patients with melioidosis, levels of IL-6, IL-10, TNF-ɑ but not IFN-γ were significantly elevated at enrollment in nonsurvivors compared with survivors [4, 16]. More recently, we have characterized the longitudinal plasma cytokine response in patients with melioidosis, including associations with survival and changes compared with uninfected controls [21]. In contrast, abundant data exist regarding the utility of IL-6 and IL-8 predicting sepsis outcomes. For example, IL-6 and IL-8 were, both individually and as part of a multibiomarker score, associated with 28-day nonsurvival in a small Spanish cohort of patients with severe sepsis or septic shock [28]. IL-8 has been included in multiple biomarker models shown to be predictive of death in both pediatric and adult patients with sepsis in North America [14, 29, 30]. Changes in IL-6 during the initial days of sepsis have also been associated with mortality in several distinct cohorts [31, 32]. IL-6 may predict organ failure in critical illness earlier than SOFA and may improve mortality discrimination of clinical judgment in patients with sepsis [33, 34]. Therefore, our findings that IL-6 and IL-8 bolster mortality prediction in patients with melioidosis are largely concordant with the published critical illness literature.

Many patients with melioidosis live in regions where resources may be limited [12]. Organ failure assessment using SOFA scores may be difficult to perform in underresourced areas, prompting evaluation using simpler clinical prediction tools [11, 35–37]. For example, calculation of the SOFA score as originally proposed requires measuring 4 blood parameters, the ability to mechanically ventilate, and the availability of vasopressor agents [38]. All of these parameters are associated with related infrastructure costs [10]. Procalcitonin and C-reactive protein, biomarkers increasingly utilized in sepsis triage, are widely available but have variable utility in Southeast Asia for infection prognosis [39, 40]. Our data suggest that a model based on plasma IL-6 and IL-8 concentrations, without clinical variables, may be a reasonable substitute for melioidosis mortality prediction models composed of clinical organ failure scores. Assays for these cytokines are not readily available in most clinical settings. However, our results may be highly relevant for the development of simple, cost-effective point-of-care assays to assist clinicians with varying expertise across a range of settings who are trying to judiciously allocate resources and optimally manage sick patients [41, 42].

Our study has several strengths. It is one of the first to include prospectively enrolled patients with melioidosis in independent cohorts from multiple sites. Culture-proven *B. pseudomallei* infection, a criterion for inclusion in this study, is the gold standard for diagnosis of melioidosis. Sample processing, tracking, and assaying were rigorously performed to reduce unmeasured confounding or batch effects. There were minimal missing data or loss to follow-up in these cohorts.

Our study also has several limitations. All subjects included were enrolled after admission to a regional medical center. Given the high patient transfer rate from outside facilities, the time of enrollment may reflect multiple days of symptoms. Our models also incorporated only a single time-point measurement, potentially limiting the utility of a SOFA score that was originally designed to be sequentially assessed [38]. Furthermore, changes in inflammatory cytokines during the first several days of illness may be more predictive of long-term outcomes than a single assessment [21, 32]. Our external validation set differed from our primary patient sets in several ways, including time of enrollment, the organ failure assessment, and the assay for cytokine concentration measurement. While bead-based multiplex and electrochemiluminescence assays differ in sensitivity ranges, comparable relative differences among samples have been previously reported [43]. However, these limitations likely contribute to the reduction in AUCs in the external validation set biomarker models. Quantifying the clinical utility of biomarker models is difficult outside of a randomized clinical trial [27]. While we used multiple methods to justify our conclusions, additional studies are needed to better define the clinical utility of our findings. Finally, future studies may consider biomarkers representiving alternative pathways to organ dysfunction [44, 45].

In summary, we developed and validated a 2-biomarker model for predicting 28-day mortality in melioidosis. By providing early information regarding clinical trajectory, this model potentially has widespread applicability in guiding management of patients with melioidosis.

## Supplementary Data

Supplementary materials are available at *Clinical Infectious Diseases* online. Consisting of data provided by the authors to benefit the reader, the posted materials are not copyedited and are the sole responsibility of the authors, so questions or comments should be addressed to the corresponding author.

ciaa126_suppl_Supplemental_Tables

## Notes

***Acknowledgments.*** The authors thank the patients and staff at Sunpasithiprasong Hospital, Udon Thani Hospital, Mukdahan Hospital, and Mahidol-Oxford Tropical Medicine Research Unit, as well as Mark Wurfel and Carmen Mikacenic.

***Financial support.*** This work was supported by the US National Institutes of Health (grant numbers T32GM086270, R01HL113382, R01AI137111, and U01AI115520) and the Wellcome Trust (grant numbers 090219/Z/09/Z and 101103/Z/13/Z).

***Potential conflicts of interest.*** The authors: No reported conflicts of interest. All authors have submitted the ICMJE Form for Disclosure of Potential Conflicts of Interest. Conflicts that the editors consider relevant to the content of the manuscript have been disclosed.
