# Supplementary material for: A 2-Biomarker Model Augments Clinical Prediction of Mortality in Melioidosis
Source: Clin Infect Dis. 2020 Feb 8;72(5):821–8. doi: 10.1093/cid/ciaa126 (PMC7935382; doi:10.1093/cid/ciaa126)
Supplement: ciaa126_suppl_Supplemental_Tables [file ciaa126_suppl_supplemental_tables.docx]

**A Two Biomarker Model Augments Clinical Prediction of Mortality in Melioidosis**

Shelton W. Wright, Taniya Kaewarpai, Lara Lovelace-Macon, Deirdre Ducken, Viriya Hantrakun, Kristina E. Rudd, Prapit Teparrukkul, Rungnapa Phunpang, Peeraya Ekchariyawat, Adul Dulsuk, Boonhthanom Moonmueangsan, Chumpol Morakot, Ekkachai Thiansukhon, Direk Limmathurotsakul, Narisara Chantratita, T. Eoin West

**Supplementary information**

Supplemental Table 1: Discrimination of mortality using clinical variables, IL-6, and IL-8

|  | **AUC^a^** | **95% CI** | **P value^b^** |
| --- | --- | --- | --- |
| **Derivation set** |  |  |  |
| IL-6 | 0.80 | 0.72-0.89 | … |
| IL-8 | 0.81 | 0.73-0.89 | … |
| IL-6 + IL-8 | 0.83 | 0.75-0.91 | 0.30 |
| Modified SOFA clinical model^c^ | 0.78 | 0.69-0.87 | ref |
| Modified SOFA + IL-6 | 0.85 | 0.78-0.92 | 0.02 |
| Modified SOFA + IL-8 | 0.85 | 0.78-0.92 | 0.03 |
| Modified SOFA + IL-6 + IL-8 | 0.86 | 0.79-0.92 | 0.01 |
| **Internal validation set** |  |  |  |
| IL-6 | 0.85 | 0.75-0.94 | … |
| IL-8 | 0.87 | 0.78-0.95 | … |
| IL-6 + IL-8 | 0.88 | 0.80-0.95 | 0.20 |
| Modified SOFA clinical model^c^ | 0.81 | 0.72-0.91 | ref |
| Modified SOFA + IL-6 | 0.90 | 0.83-0.97 | 0.03 |
| Modified SOFA + IL-8 | 0.89 | 0.82-0.96 | 0.04 |
| Modified SOFA + IL-6 + IL-8 | 0.91 | 0.84-0.97 | 0.03 |
| **External validation set** |  |  |  |
| IL-6 | 0.75 | 0.67-0.82 | … |
| IL-8 | 0.78 | 0.71-0.85 | … |
| IL-6 + IL-8 | 0.78 | 0.71-0.85 | 0.67 |
| Organ failure clinical model^d^ | 0.76 | 0.68-0.84 | ref |
| Organ failure + IL-6 | 0.79 | 0.73-0.86 | 0.14 |
| Organ failure + IL-8 | 0.80 | 0.74-0.87 | 0.04 |
| Organ failure + IL-6 + IL-8 | 0.81 | 0.74-0.88 | 0.02 |

^a^ AUC: Area under the receiver operating characteristic curve.

^b^ P value for comparison of biomarker model to the clinical variable model for each set (modified SOFA clinical model or organ failure clinical model).

^c^ Modified SOFA clinical model includes age, sex, Charlson Comorbidity Index, and modified SOFA score.

^d^ Organ failure clinical model includes age, sex, Charlson Comorbidity Index, and respiratory failure or shock.

Supplemental Table 2: Discrimination of mortality comparing the addition of IL-6 and IL-8 to a clinical variable model

^a^ Modified SOFA clinical model includes age, sex, Charlson Comorbidity Index and modified SOFA score.

| **Set** | **Model** | **AUC^b^ (95% CI)** | **IDI^c^** |
| --- | --- | --- | --- |
| **Derivation** | Modified SOFA^a^ | 0.78 (0.69-0.87) | ref |
|  | Modified SOFA^a^ + IL-6 & IL-8 | 0.86 (0.79-0.92) | 0.15 ± 0.34 P<0.0001 |
| **Internal validation** | Modified SOFA^a^ | 0.81 (0.72-0.91) | ref |
|  | Modified SOFA^a^ + IL-6 & IL-8 | 0.89 (0.82-0.97) | 0.23 ± 0.05 P<0.0001 |
| **External validation** | Organ failure^d^ | 0.76 (0.68-0.84) | ref |
|  | Organ failure^d^ + IL-6 & IL-8 | 0.81 (0.74-0.88) | 0.06 ± 0.02 P=0.001 |

^b^ AUC: Area under the receiver operating characteristic curve.

^c^  IDI: Integrated discrimination improvement; values represent estimates of improvement in clinical variable model discrimination, ± standard error, following addition of biomarkers.

^d^ Organ failure clinical model includes age, sex, Charlson Comorbidity Index, and respiratory failure or shock.

Supplemental Table 3: Association of IL-6 and IL-8 with 28-day mortality in the internal validation set

| **Biomarker**  **(log_10_)** | **Unadjusted** | | | **Adjusted^a^** | | | **Modified SOFA-adjusted^b^** | | |
| --- | --- | --- | --- | --- | --- | --- | --- | --- | --- |
|  | OR | 95% CI | P value | OR | 95% CI | P value | OR | 95% CI | P value |
| **IL-6** | 8.91 | 3.53-22.5 | <0.001 | 14.40 | 4.65-44.32 | <0.001 | 10.27 | 3.13-33.65 | <0.001 |
| **IL-8** | 6.54 | 2.85-15.00 | <0.001 | 8.33 | 3.25-21.39 | <0.001 | 6.64 | 2.34-18.84 | <0.001 |

^a^ For adjusted model, P values were determined using logistic regression adjusted for age, sex and Charlson Comorbidity Index.

^b^ For modified SOFA-adjusted model, P values were determined using logistic regression adjusted for age, sex, Charlson Comorbidity Index and modified SOFA score.

Supplemental Table 4: Mortality prediction using clinical variables with or without IL-6 and IL-8 in the internal validation set

| **Model** | **Variable** | **OR** | **95% CI** | **P value** |
| --- | --- | --- | --- | --- |
| **Clinical variables** | Age | 0.97 | 0.91-1.03 | 0.31 |
|  | Female sex | 0.87 | 0.25-2.98 | 0.82 |
|  | Charlson Comorbidity Index | 1.66 | 0.96-2.88 | 0.07 |
|  | Modified SOFA | 1.45 | 1.20-1.76 | <0.001 |
| **Clinical variables + IL-6^a^** | Age | 0.95 | 0.88-1.03 | 0.21 |
|  | Female sex | 0.87 | 0.21-3.67 | 0.85 |
|  | Charlson Comorbidity Index | 2.41 | 1.17-4.97 | 0.01 |
|  | Modified SOFA | 1.17 | 0.93-1.48 | 0.18 |
|  | IL-6 | 10.3 | 3.13-33.65 | <0.001 |
| **Clinical variables + IL-8^b^** | Age | 0.96 | 0.89-1.04 | 0.31 |
|  | Female sex | 0.97 | 0.24-3.96 | 0.97 |
|  | Charlson Comorbidity Index | 2.09 | 1.04-4.22 | 0.04 |
|  | Modified SOFA | 1.12 | 0.87-1.43 | 0.38 |
|  | IL-8 | 6.64 | 2.34-18.84 | <0.001 |

^a^ Model containing clinical variables and log_10_ IL-6 differed significantly by likelihood ratio test (P=2 x 10^-6^) compared to the clinical variable model.

^b^ Model containing clinical variables and log_10_ IL-8 differed significantly by likelihood ratio test (P=1.5 x 10^-5^) compared to the clinical variable model.

| **Biomarker** | **Unadjusted** | | | **Adjusted^a^** | | | **Organ failure-adjusted^b^** | | |
| --- | --- | --- | --- | --- | --- | --- | --- | --- | --- |
|  | OR | 95% CI | P value | OR | 95% CI | P value | OR | 95% CI | P value |
| **IL-6** | 4.18 | 2.32-7.52 | <0.001 | 4.06 | 2.21-7.44 | <0.001 | 2.51 | 1.36-4.60 | 0.003 |
| **IL-8** | 5.87 | 3.11-11.10 | <0.001 | 5.73 | 3.07-10.68 | <0.001 | 3.35 | 1.65-6.79 | 0.001 |

Supplemental Table 5: Association of IL-6 or IL-8 with 28-day mortality in the external validation set

^a^ For adjusted model, P values were determined using logistic regression adjusted for age, sex, and Charlson Comorbidity Index.

^b^ For organ failure-adjusted model, P values were determined using logistic regression adjusted for age, sex, Charlson Comorbidity Index, and cardiopulmonary organ failure

Supplemental Table 6: Mortality prediction using clinical variables with or without IL-6 and IL-8 in the external validation set

| **Model** | **Variable** | **OR** | **95% CI** | **P value** |
| --- | --- | --- | --- | --- |
| **Clinical variables** | Age | 1.01 | 0.97-1.04 | 0.62 |
|  | Female sex | 0.62 | 0.28-1.38 | 0.24 |
|  | Charlson Comorbidity Index | 1.05 | 0.84-1.31 | 0.69 |
|  | Organ failure**^a^** | 7.47 | 3.63-15.39 | <0.001 |
| **Clinical variables + IL-6^b^** | Age | 1.01 | 0.97-1.04 | 0.77 |
|  | Female sex | 0.66 | 0.29-1.50 | 0.32 |
|  | Charlson Comorbidity Index | 1.05 | 0.84-1.31 | 0.66 |
|  | Organ failure**^a^** | 4.58 | 2.09-10.05 | <0.001 |
|  | IL-6 | 2.51 | 1.33-4.72 | 0.005 |
| **Clinical variables + IL-8^c^** | Age | 1.01 | 0.97-1.04 | 0.68 |
|  | Female sex | 0.65 | 0.28-1.47 | 0.30 |
|  | Charlson Comorbidity Index | 1.02 | 0.81-1.28 | 0.87 |
|  | Organ failure**^a^** | 3.38 | 1.45-7.87 | 0.005 |
|  | IL-8 | 3.35 | 1.60-7.01 | 0.001 |

^a^ Organ failure is defined as the occurrence of respiratory failure requiring invasive mechanical ventilation or shock requiring an infusion of epinephrine, norepinephrine, dopamine or dobutamine.

^b^ Model containing clinical variables and log_10_ IL-6 differed significantly by likelihood ratio test (P=0.002) compared to the clinical variable model.

^c^ Model containing clinical variables and log_10_ IL-8 differed significantly by likelihood ratio test (P=8x10^-4^) compared to the clinical variable model.

**Supplemental Figure 1: Receiver operating characteristic curves for discrimination of mortality using IL-6**

A. Derivation set B. Internal validation set

C. External validation set

Receiver operating characteristic curves are shown for the baseline risk (age, sex, Charlson Comorbidity Index) model, baseline risk + modified SOFA model, and baseline risk + modified SOFA + IL-6 model for the derivation set (A) (AUCs=0.63, 0.78, and 0.85, respectively) and for the internal validation set (B) (AUCs=0.56, 0.81, and 0.90, respectively). Receiver operating characteristic curves are shown for the baseline risk (age, sex, Charlson Comorbidity Index) model, baseline risk + cardiopulmonary organ failure (respiratory failure requiring invasive mechanical ventilation or shock requiring an infusion of epinephrine, norepinephrine, dopamine or dobutamine) model, and baseline risk + cardiopulmonary organ failure + IL-6 model in the external validation set (C) (AUC=0.58, 0.76, and 0.79, respectively).

**Supplemental Figure 2: Receiver operating characteristic curves for discrimination of mortality using IL-8**

A. Derivation set B. Internal validation set

C. External validation set

Receiver operating characteristic curves are shown for the baseline risk (age, sex, Charlson Comorbidity Index) model, baseline risk + modified SOFA model, and baseline risk + modified SOFA + IL-6 model for the derivation set (A) (AUCs=0.63, 0.78, and 0.85, respectively) and for the internal validation set (B) (AUCs=0.56, 0.81, and 0.89, respectively). Receiver operating characteristic curves are shown for the baseline risk (age, sex, Charlson Comorbidity Index) model, baseline risk + cardiopulmonary organ failure (respiratory failure requiring invasive mechanical ventilation or shock requiring an infusion of epinephrine, norepinephrine, dopamine or dobutamine) model, and baseline risk + cardiopulmonary organ failure + IL-6 model in the external validation set (C) (AUC=0.58, 0.76, and 0.80, respectively).
